# Longitudinal Analyses of Childhood Growth: Evidence From Project Koshu

**DOI:** 10.2188/jea.JE20140130

**Published:** 2015-01-05

**Authors:** Kohta Suzuki

**Affiliations:** Department of Health Sciences, Interdisciplinary Graduate School of Medicine and Technology, University of Yamanashi, Chuo, Yamanashi, Japan; 山梨大学大学院医学工学総合研究部 社会医学講座

**Keywords:** smoking, pregnancy, fetal growth, childhood growth, multilevel analysis

## Abstract

Recently, it has been suggested that fetal and infant environments are associated with childhood and adulthood health status, specifically regarding presence of obesity and chronic diseases. This concept is known as the “Developmental Origins of Health and Disease (DOHaD) hypothesis.” Thus, it is necessary to collect information about the fetal and infancy periods in order to examine the association between fetal and infancy exposures and later growth. Based on the DOHaD hypothesis, childhood growth trajectories, which were described by multilevel analysis, might be important in examining the effects of early-life environment on later-life health. The author and colleagues examined the association between maternal smoking during pregnancy and fetal/childhood growth, specifically risk of childhood obesity, by using the dataset from an ongoing prospective cohort study called “Project Koshu,” which enrolled pregnant women and their children from a rural area of Japan. Children born to smoking mothers were likely to have lower birth weights and, thereafter, to show an increase in body mass index compared to children of non-smoking mothers. Differences in pubertal growth patterns by gender and childhood weight status were then examined. Growth rate and height gain trajectories were similar between genders, although pubertal growth spurts were observed earlier in girls than in boys. The overweight/obese children grew faster than did the non-overweight children in the early pubertal stages, and the non-overweight children caught up and showed greater height gains at older ages. Because Project Koshu is ongoing, further studies examining new research questions will be conducted with larger sample sizes.

## Abstract (ja)

近年、胎児期あるいは幼児期の環境がその後の発育、特に、肥満や生活習慣病などの健康状態に影響すると示唆されており、この概念は「Developmental Origins of Health and Disease (DOHaD) 説」として知られている。胎児期や幼児期における曝露因子とその後の発育を検討するためには、胎児期からの情報を収集することが必須である。一方で、「ライフコース疫学」という言葉もよく用いられるようになってきた。DOHaD説に基づいて、マルチレベル解析を用いて子どもの発育を軌跡として記述することは、胎児期や出生後早期の影響を検討するために重要である。著者らは妊娠中の喫煙と、胎児期また小児期の発育、特に肥満との関係を、現在も継続して実施されている、妊娠期からの前向きコホート研究「甲州プロジェクト」のデータを用いて検討した。その結果、妊娠中に喫煙している母親から生まれた児は出生体重が小さくなり、さらにその後Body Mass Index（BMI）が増加しやすい傾向を示した。また、思春期の発育パターンが、男女で、また体格によって異なることも検討した。身長の伸びなどの発育パターンは男女で同様であったが、女児で男児よりも思春期の身長の伸びのピークが早いことが観察された。また、過体重あるいは肥満の児は、非肥満の児に比べ発育のピークが早いこと、非肥満の児は中学生の時期により身長が伸びることが示された。甲州プロジェクトは継続しており、今後、胎児期から小児期にかけての新しい研究テーマについても、対象者数を現在より増やして検討することが可能である。

## INTRODUCTION

Recently, the Developmental Origins of Health and Disease (DOHaD) hypothesis has been suggested.^1^ Similar concepts, which describe the association between a specific path of growth—consisting of slow growth in fetal life and rapidly increasing body mass index (BMI) as an infant—and the development of adulthood chronic diseases, were previously known as fetal programming and Barker’s hypothesis.^1^^–^^5^ Examining these hypotheses and concepts will require descriptions of the study participants from the fetal period. However, this information has been difficult to collect in a timely matter in most birth cohort studies, as participants in these studies are usually recruited after birth and information on the prenatal period (eg, maternal lifestyle habits during pregnancy) is collected retrospectively, leading to potential information biases and measurement errors.

Because it is important to obtain accurate descriptions of maternal and child health status to minimize such biases and errors, recent studies like the Japan Environment and Children’s Study have begun recruiting participants during the early prenatal period.^6^ Prior to these recent studies, an ongoing prospective cohort study of pregnant women and their children, called Project Koshu, was initiated in a rural area of Japan. Although this study has several limitations (eg, a relatively small sample size), some researchers have examined the association between fetal environment and childhood growth using the dataset of this study.^7^^–^^17^ For instance, the relationship between maternal smoking during pregnancy and childhood growth, especially as it pertains to childhood obesity, was examined.^7^^,^^9^^–^^11^ The present review introduces the prospective Project Koshu study and some of its findings.

## BRIEF INTRODUCTION TO PROJECT KOSHU

The Koshu City (formerly Enzan City) administration office and the Department of Health Sciences, Interdisciplinary Graduate School of Medicine and Technology, University of Yamanashi cooperatively conduct Project Koshu, an ongoing prospective cohort study of pregnant women and their children that commenced in 1988. The population of Koshu City is 33 000, with approximately 200 births each year. We expected a high follow-up rate in this project because most of the children do not migrate elsewhere until graduation from junior high school.

**Figure 1.**
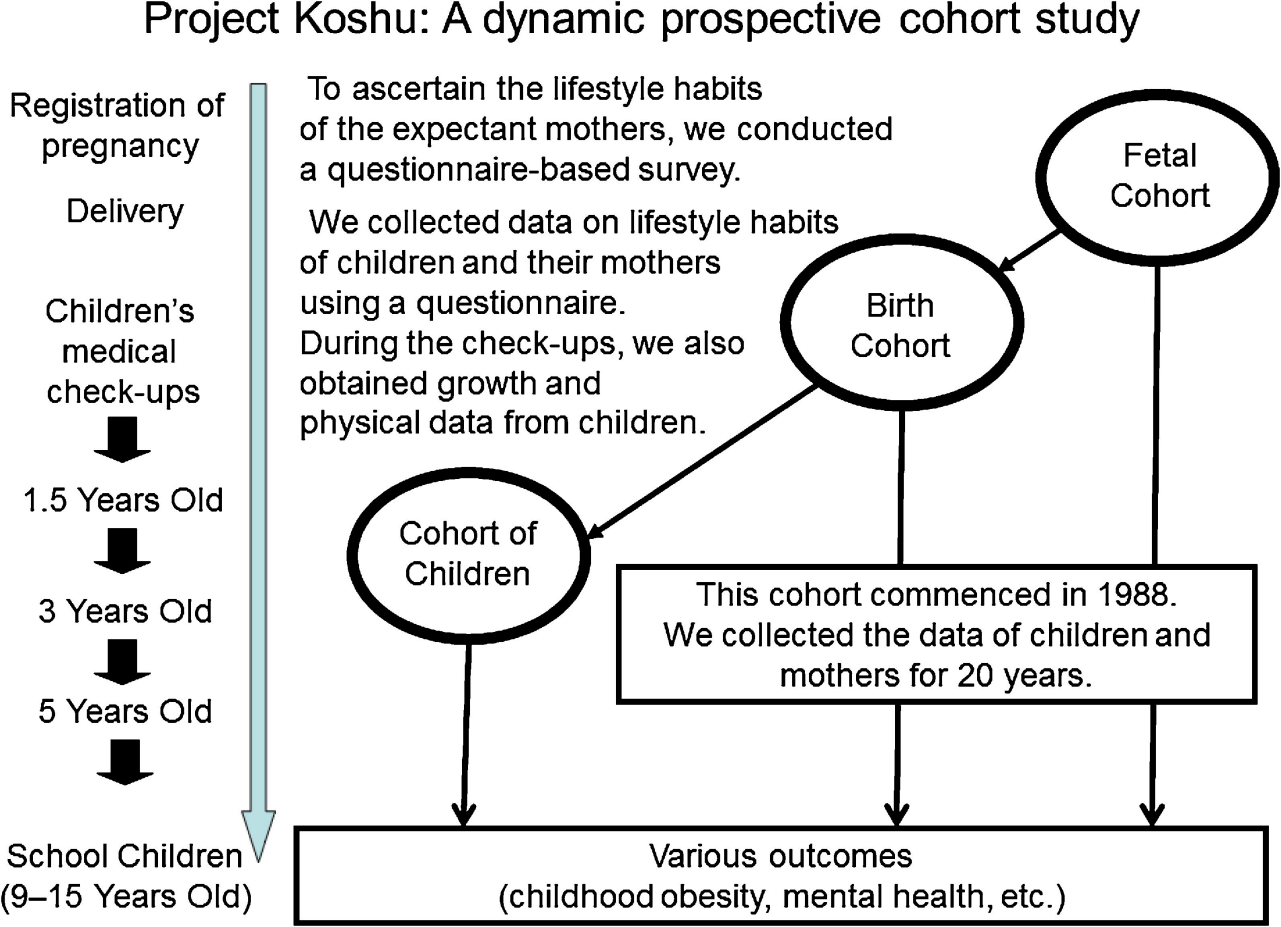
Brief study design of Project Koshu

Originally, the purpose of the study was to describe the current status of maternal and child health in the area. For example, a trend of maternal smoking during pregnancy was previously reported.^14^ Then, after accumulating the data from each year, longitudinal datasets were created to examine the association between exposures in fetal and infant periods, such as maternal smoking during pregnancy or childhood sleep duration, and childhood growth and development. Thus, depending on the research question, various cohorts were able to be established. The scheme of Project Koshu is presented in Figure 1.

In Japan, pregnant women are encouraged to register their pregnancy at the city office, and children are invited to undergo a medical checkup at ages 1.5, 3, and 5 years. First, to ascertain the lifestyle habits of expectant mothers, we conducted a questionnaire-based survey with expectant mothers who visited the city office to register their pregnancies. In the study area, over 80% of expectant mothers registered their pregnancy in the first trimester, and almost all registered by 18 gestational weeks. Next, at each medical checkup of the children born to these mothers, we surveyed the lifestyle habits of the children and their mothers using a questionnaire and collected data on children’s growth and physical characteristics. Subsequently, we collected anthropometric data from elementary and junior high school children, which are measured annually in April for each grade, in accordance with the Japanese School Health and Safety Law. In this manner, childhood anthropometric data were repeatedly obtained.

In order to ensure confidentiality, the mothers and children were identified by unique numbers to match the data obtained from the initial pregnancy survey and the later medical checkups. This study was approved by the Ethical Review Board of the University of Yamanashi School of Medicine and was conducted in accordance with the Guidelines Concerning Epidemiological Research (issued by the Ministry of Education, Culture, Sports, Science, and Technology and the Ministry of Health, Labour and Welfare, Japan). Written informed consent from the participants was obtained prior to administration of the initial survey.

## EFFECT OF MATERNAL SMOKING DURING PREGNANCY ON FETAL AND CHILDHOOD GROWTH

Maternal smoking during pregnancy is a major cause of low birth weight and intrauterine growth restriction.^18^^–^^21^ This association has been confirmed in this area.^8^ In addition, it has been suggested that maternal smoking cessation before or during early pregnancy may still allow for appropriate fetal and childhood growth.^13^

However, because few studies have examined the effect of maternal smoking during pregnancy on childhood growth and development in Japan, Mizutani et al examined the association between maternal lifestyle factors, including smoking during pregnancy, and childhood obesity.^7^ This may have been the first article to examine the effect of maternal smoking on childhood obesity in Japan. Maternal smoking was significantly associated with overweight (adjusted odds ratio [OR] 2.2, 95% confidence interval [CI] 1.1–4.1) and obesity (adjusted OR 3.9, 95% CI 1.5–10.6) among 5-year-old children.^7^

We then examined whether the association between maternal smoking and overweight or obesity persists up to 9–10 years of age.^9^ In this analysis, maternal smoking was associated with overweight at the age of 9–10 years (adjusted OR 1.9, 95% CI 1.03–3.5). Furthermore, children whose mothers had smoked during early pregnancy exhibited an independently elevated risk for obesity compared with those whose mothers had not smoked or had quit smoking (adjusted OR 2.6, 95% CI 1.02–6.4). However, these point estimates at the age of 9–10 years were considerably lower than those observed at 5 years.

Next, because there were some differences between children at 5 years of age and children at 9–10 years of age in adjusted ORs for the association between maternal smoking during pregnancy and childhood obesity/overweight, we examined the association between maternal smoking during pregnancy and being overweight in childhood during different periods using two cohorts from the same population: a birth cohort (the first cohort) and non-overweight children at 5 years of age (the second cohort).^12^ We found an association between maternal smoking during pregnancy and being overweight only in male children in the first cohort analysis (adjusted OR 4.5, 95% CI 2.0–10.2). On the other hand, there was no significant association between maternal smoking during pregnancy in the second cohort analysis. These results suggest that the effects of maternal smoking during pregnancy on overweight in childhood tend to manifest before 5 years of age, especially in male children.^12^

Maternal smoking during pregnancy can result in the birth of an undernourished baby. Such prenatal nutritional deprivation may lead to increased nutrient absorption and resultant postnatal obesity. It has been reported that undernutrition during pregnancy increases the risk of adult obesity,^22^ causes intrauterine growth retardation, and increases the risk of abnormal glucose tolerance.^23^ Our results were consistent with these reports. One study found that both duration and quantity of smoking were positively associated with childhood obesity in a dose-dependent manner.^24^ Further, not all women who smoked during early pregnancy continued to smoke up to delivery in this study.^24^ The group of women who smoked during early pregnancy consisted of women who quit during early pregnancy, those who quit during late pregnancy, and those who continued smoking up to delivery. Therefore, it was suggested that our results might be underestimated.

## DESCRIPTION OF CHILDHOOD GROWTH TRAJECTORY: EXAMPLES OF MULTILEVEL ANALYSIS

The term “life-course epidemiology” has recently become popular. As previously described, Barker’s hypothesis and the DOHaD hypothesis are probably the best-known examples of life-course epidemiology. Because these hypotheses state that poor fetal nutrition, indicated by small birth size, leads to fetal adaptations that alter the propensity to adult diseases,^25^ it is necessary to describe the growth trajectories during childhood in order to assess the veracity of these claims. However, no study has conducted such an analysis to clarify the association between maternal lifestyle during pregnancy, which is used as a proxy indicator of fetal environment, and childhood growth or development. Twisk stated that multilevel analysis is usually suitable for analyzing correlated data.^26^ Therefore, individual growth analysis, which includes both individual and age as different-level variables, is appropriate for data with repeated measurements obtained in Project Koshu to describe the growth trajectories during childhood. Further, gender differences in fetal growth may be the basis for gender differences in the sensitivity to fetal programming^27^; however, such differences have not been studied previously.

**Figure 2.**
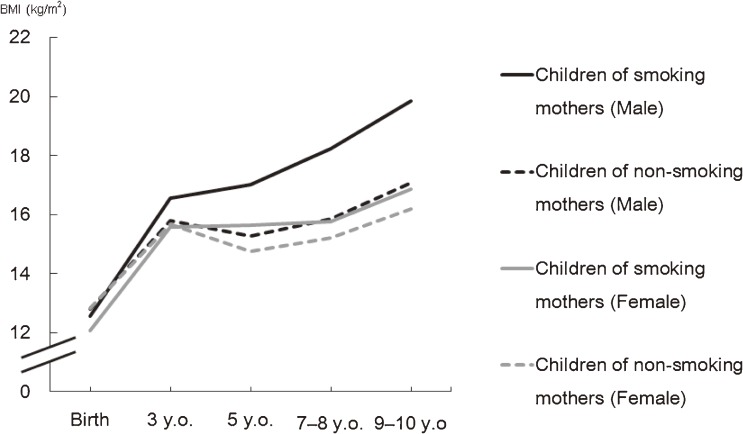
Children’s body mass index (BMI) trajectories by maternal smoking status during pregnancy, calculated using individual growth analysis (original data: Suzuki et al, 2011)

We examined the gender differences in the association between maternal smoking during pregnancy and later growth in childhood by conducting a multilevel analysis (using a fixed-effects model).^10^ The mean birth weight of both male and female children whose mothers had smoked during pregnancy was significantly lower than the birth weights of children born to non-smoking mothers (*P* < 0.01). Childhood BMI at each subsequent check-up age significantly increased only among male children born to smoking mothers. Further, this increase was continuously observed after 3 years of age (Figure 2). The results of a BMI z-score analysis were also similar to these BMI analyses. Moreover, a random-effects hierarchical linear regression model was used to examine the same association.^11^ In this model, there was very strong evidence that the effect of age (in months) on the increase in BMI z-scores for the male children was enhanced by maternal smoking during pregnancy (*P* < 0.0001). In contrast, only weak evidence was found among female children for an interaction between age in months and maternal smoking during pregnancy (*P* = 0.054), suggesting that the effect of maternal smoking during pregnancy on children’s early-life BMI trajectories differed by gender. It has been suggested that male children are likely to be more vulnerable to adverse environmental factors, such as exposure to smoking.^28^ In addition, prenatal exposure to nicotine increases testosterone levels in rat fetuses,^29^ and it has been suggested that androgens play an important role in the regulation of body fat distribution.^30^ Thus, our results may be consistent with these biological explanations.

Zheng et al described gender-based height growth patterns in Japanese school children using a multilevel analysis, as determining standard pubertal growth patterns using longitudinal anthropometric measures is important in growth assessment.^15^ Height was similar between genders at 6.5–9.5 years of age, but girls grew faster and were taller than boys at 10.5–11.5 years of age. Subsequently, boys caught up and exceeded girls’ heights starting at age 12.5. Height gain trajectories showed that the girls’ annual height gains increased slowly and peaked from 9.5 to 11.5 years of age, while boys’ height gains declined slightly at first and peaked at 11.5–12.5 years of age. The gender-based differences in height gains were significant from 7.5–14.5 years of age (*P* < 0.0001). Growth rate and height gain trajectories were similar between genders, although pubertal growth spurts were observed earlier in girls than in boys. These findings were similar to the results of a recent cross-sectional study in Croatia^31^ and the results of a Japanese national survey.^32^ Compared with these results, the peak of the annual difference in median height (a similar meaning to annual height gain), which was described in the World Health Organization Multicentre Growth Reference Study, occurred 1 year later in boys but was almost the same age in girls.^33^

Zheng et al also examined the differences in growth patterns during adolescence between overweight/obese and non-overweight children in Japan.^16^ Overweight/obese girls grew taller in the first-half period of primary school and junior high school, reached their peak height gain about a year earlier than non-overweight girls did, and experienced an earlier decrease in height gain. Similarly, overweight/obese boys initially gained more height than non-overweight boys did. Additionally, non-overweight boys maintained a higher rate of height gain from the age of peak height gain, although the age of peak height gain did not differ between the two groups.

Wronka assessed the pubertal height gain of girls in four different weight categories.^34^ The results indicated that girls who were overweight or obese at 7 years of age showed a rapid growth rate. They gained more height than their non-overweight peers between 7 and 9 years of age, and thereafter, their annual height gain was lower than that of their non-overweight peers. This crossover in height gain was consistent with the results from Project Koshu. In addition, according to a review, most other studies supported the finding that obesity is associated with a rapid progression of pubertal growth in girls.^35^ Two studies using stature growth indicators also suggested that childhood BMI was related to an earlier age of peak height velocity.^36^^,^^37^ Their results for girls were also consistent with the findings of Zheng et al.

In contrast, previous reports regarding the association between prepubertal BMI and adolescent growth among boys remain controversial. It has been suggested that boys with a higher prepubertal BMI had an earlier age of peak height velocity than boys with lower prepubertal BMI.^36^^,^^37^ Buyken et al reported that childhood body composition is associated with the progression of puberty, but not the timing of the take-off age for pubertal growth, in German children.^38^ Further, a recent cohort study showed that boys with a high BMI z-score during childhood experienced a late pubertal onset.^39^ Zheng et al suggested that overweight/obese boys gain more height annually in early stages and less height thereafter than non-overweight boys. This finding might suggest that overweight/obese boys also experience an earlier pubertal growth spurt. However, no significant differences in the age of peak annual height gain between the two BMI groups were identified in boys.

## CONCLUSION AND FUTURE IMPLICATIONS

In conclusion, although Project Koshu is a small prospective cohort study in a rural area of Japan, important evidence regarding the relationship between maternal and child health, particularly in the association between maternal smoking during pregnancy and childhood growth, has been reported. The strength of this cohort study is the ability to examine the association between fetal environment, which was defined as the lifestyle habits of pregnant women, and children’s growth. In addition, because relatively advanced statistical methods, like multilevel analysis, were applied to this dataset consisting of repeatedly measured anthropometric data, childhood growth trajectories were appropriately described. These results are important not only in establishing public health strategies in the community but also in exploring the mechanisms of fetal programming, Barker’s hypothesis, and the DOHaD hypothesis. Because the study is ongoing, further studies will be able to explore new research questions with larger sample sizes and various statistical methods. For example, the pathways between maternal smoking during pregnancy and rapid growth in infancy have recently been examined using a covariance structure analysis.^17^ In addition, it is important to consider how to convey the results to the community in the future.

## ONLINE ONLY MATERIAL

Abstract in Japanese.
