# Supplementary material for: Longitudinal Analyses of Childhood Growth: Evidence From Project Koshu
Source: J Epidemiol. 2015 Jan 5;25(1):2–7. doi: 10.2188/jea.JE20140130 (PMC4275431; doi:10.2188/jea.JE20140130)
Supplement: Abstract in Japanese. [file je-25-002-s001.pdf]

子どもの発育に関する縦断的解析：甲州プロジェクトからの知見

鈴木　孝太

山梨大学大学院医学工学総合研究部　社会医学講座

近年、胎児期あるいは幼児期の環境がその後の発育、特に、肥満や生活習慣病などの健康状態に影響すると示唆されており、この概念は「Developmental Origins of Health and Disease (DOHaD) 説」として知られている。胎児期や幼児期における曝露因子とその後の発育を検討するためには、胎児期からの情報を収集することが必須である。一方で、「ライフコース疫学」という言葉もよく用いられるようになってきた。DOHaD 説に基づいて、マルチレベル解析を用いて子どもの発育を軌跡として記述することは、胎児期や出生後早期の影響を検討するために重要である。著者らは妊娠中の喫煙と、胎児期また小児期の発育、特に肥満との関係を、現在も継続して実施されている、妊娠期からの前向きコホート研究「甲州プロジェクト」のデータを用いて検討した。その結果、妊娠中に喫煙している母親から生まれた児は出生体重が小さくなり、さらにその後 Body Mass Index（BMI）が増加しやすい傾向を示した。また、思春期の発育パターンが、男女で、また体格によって異なることも検討した。身長の伸びなどの発育パターンは男女で同様であったが、女児で男児よりも思春期の身長の伸びのピークが早いことが観察された。また、過体重あるいは肥満の児は、非肥満の児に比べ発育のピークが早いこと、非肥満の児は中学生の時期により身長が伸びることが示された。甲州プロジェクトは継続しており、今後、胎児期から小児期にかけての新しい研究テーマについても、対象者数を現在より増やして検討することが可能である。

キーワード：喫煙、妊娠、胎内発育、小児発育、マルチレベル解析
